# Pyramiding of Low Chalkiness QTLs Is an Effective Way to Reduce Rice Chalkiness

**DOI:** 10.1186/s12284-023-00680-x

**Published:** 2024-01-08

**Authors:** Weifeng Yang, Songliang Chen, Qingwen Hao, Haitao Zhu, Quanya Tan, Shaojun Lin, Guodong Chen, Zhan Li, Suhong Bu, Zupei Liu, Guifu Liu, Shaokui Wang, Guiquan Zhang

**Affiliations:** 1 Guangdong Provincial Key Laboratory of Plant Molecular Breeding, State Key Laboratory for Conservation and Utilization of Subtropical Agro-Bioresources, South China Agricultural University, Guangzhou, 510642 China; 2 Guangdong Laboratory for Lingnan Modern Agriculture, Guangzhou, China

**Keywords:** Rice chalkiness, High temperature, QTL pyramiding, Epistasis, Rice quality, Breeding

## Abstract

Rice chalkiness is a key limiting factor of high-quality rice. The breeding of low chalkiness varieties has always been a challenging task due to the complexity of chalkiness and its susceptibility to environmental factors. In previous studies, we identified six QTLs for the percentage of grain chalkiness (PGC), named *qPGC5*, *qPGC6*, *qPGC8.1*, *qPGC8.2*, *qPGC9* and *qPGC11*, using single-segment substitution lines (SSSLs) with genetic background of Huajingxian 74 (HJX74). In this study, we utilized the six low chalkiness QTLs to develop 17 pyramiding lines with 2–4 QTLs. The results showed that the PGC decreased with the increase of QTLs in the pyramiding lines. The pyramiding lines with 4 QTLs significantly reduced the chalkiness of rice and reached the best quality level. Among the six QTLs, *qPGC5* and *qPGC6* showed greater additive effects and were classified as Group A, while the other four QTLs showed smaller additive effects and were classified as Group B. In pyramiding lines, although the presence of epistasis, additivity remained the main component of QTL effects. *qPGC5* and *qPGC6* showed stronger ability to reduce rice chalkiness, particularly in the environment of high temperature (HT) in the first cropping season (FCS). Our research demonstrates that by pyramiding low chalkiness QTLs, it is feasible to develop the high-quality rice varieties with low chalkiness at the best quality level even in the HT environment of FCS.

**Supplementary Information:**

The online version contains supplementary material available at 10.1186/s12284-023-00680-x.

## Background

Rice (*Oryza sativa* L.) is a vital food crop for the world’s population (Fukagawa and Ziska 2019). Rice chalkiness is characterized by the white opaque area in the kernel, which usually exhibits poorer appearance performance and negatively impacts on milling and cooking quality, consequently affecting consumer acceptability and rice market value (Fitzgerald et al. 2009; Fan et al. 2022; Li et al. 2022). By comparing the main quality traits of 452 *indica* varieties released in China from 2000 to 2014, it was observed that the proportion of varieties meeting the grade 3 national standard of rice quality for rice chalkiness was lower than that for other rice quality traits (Feng et al. 2017). Therefore, reducing rice chalkiness has become an important target in breeding of high-quality rice.

Chalkiness is a complex trait governed by multiple QTLs and is easily influenced by environmental factors. In the past decades, numerous QTLs for chalkiness have been identified in rice genome using diverse mapping populations (Sreenivasulu et al. 2015; Fan et al. 2022; Zhao et al. 2022). These QTLs, including *qPCG1*, *qWCR4*, *qWCR7*, *qPGWC-7*, *qPGWC-8*, *qACE9* and *qPCG10*, were fine-mapped on chromosomes 1, 4, 7, 8, 9 and 10, respectively (Zhou et al. 2009; Guo et al. 2011; Gao et al. 2016; Mei et al. 2018; Zhu et al. 2018; Wu et al. 2021; Shi et al. 2022). *Chalk5*, a major QTL controlling chalkiness, was firstly cloned on chromosome 5. Most rice varieties with Zhenshan 97 allele showed higher levels of *Chalk5* expression, which is correlated with increased grain chalkiness. The rare allele *chalk5* for lower chalkiness from H94 variety is beneficial for the breeding of high-quality rice varieties (Li et al. 2014). *WCR1* is a negative regulator of grain chalkiness. The *WCR1*^*A*^ allele was identified in Beilu130 variety, which reduced grain chalkiness by promoting the transcription of *WCR1* (Wu et al. 2022). Although many QTLs for chalkiness were identified, few of them have been used to improve rice quality through QTL pyramiding.

High temperature (HT) is a major environmental factor influencing rice chalkiness. HT stress during the grain-filling stage disturbs the endosperm development, resulting in an accelerated occurrence of chalkiness (Nevame et al. 2018). Some QTLs for chalkiness caused by HT were identified in HT sensitive varieties, promoting the formation of chalkiness under HT stress (Tabata et al. 2007; Wada et al. 2015; Yang et al. 2021a). These findings revealed the genetic basis that most rice varieties exhibit high chalkiness under HT conditions. In the double-cropping rice region of southern China, the higher air temperature in first cropping season (FCS) than in second cropping season (SCS) during rice grain-filling stage leads to a higher occurrence of rice chalkiness in FCS (Cheng et al. 2005; Yang et al. 2021a). Recently, in the double-cropping environments, six QTLs, *qPGC5*, *qPGC6*, *qPGC8.1*, *qPGC8.2*, *qPGC9* and *qPGC11*, for rice chalkiness were fine-mapped, showing different response to HT (Yang et al. 2021a, b, 2022). Two major QTLs, *qPGC5* and *qPGC6*, showed higher additive effects enhanced by HT, which are rare QTLs for more effectively reducing rice chalkiness under HT of FCS (Yang et al. 2022). These findings provide new genetic resources to improve rice chalkiness even under HT condition.

Complex traits of rice are regulated by epistasis interaction among QTLs. Epistasis refers to the interactions between two or more loci in genome-wide (Mackay 2014; Misra et al. 2021). Eshed and Zamir (1996) found that epistasis was less-than-additive in tomato, meaning that the effect of the combination of double loci was smaller than the sum of the effects of the corresponding individual locus. Epistasis induces hidden genetic variation for quantitative traits in different populations, making the relationship between genotype and phenotype complex and resulting in small additive effects (Carlborg and Haley 2004; Mackay 2014). It has been established that epistasis is a common feature of the genetic architecture of quantitative traits and has been detected in many quantitative traits, which indicates that epistasis is an important regulator influencing quantitative traits (Zhou et al. 2020; Kato and Horibata 2022; Tan et al. 2022a).

Over the past 20 years, single-segment substitution lines (SSSLs) in the genetic background of Huajingxian 74 (HJX74) were widespread in dissecting QTLs for important agronomic traits in rice (Xi et al. 2006; Liu et al. 2008; Wang et al. 2012a; Tan et al. 2021; Zhang 2021; Zhan et al. 2022). Then, the SSSLs carrying the desirable QTL alleles have been used to improve targeted complex traits by QTL pyramiding breeding (Guo et al. 2016; Luan et al. 2019; Zhang 2021; Tan et al. 2022a). Recently, six QTLs for percentage of grain chalkiness (PGC), named *qPGC5*, *qPGC6*, *qPGC8.1*, *qPGC8.2*, *qPGC9* and *qPGC11*, in rice were fine-mapped in the SSSLs (Yang et al. 2021a, b, 2022). The six QTLs for low chalkiness were utilized in the present study to develop pyramiding lines with different QTL combinations. The results showed that the PGC significantly reduced with increase of QTLs in the pyramiding lines, and the combinations of 3–4 QTLs showed the stable ability to reduce chalkiness. The combination of *qPGC5* and *qPGC6* displayed more efficiency in reducing chalkiness even under HT conditions. Our research demonstrates that the rice chalkiness can be reduced by using these QTLs even under HT condition of FCS.

## Materials and Methods

### Plant Materials and Field Experiments

Six SSSLs (22 − 05, 15 − 06, 15 − 08, 03–08, 11 − 09 and HP67-11) with HJX74 genetic background, carrying *qPGC5*, *qPGC6*, *qPGC8.1*, *qPGC8.2*, *qPGC9* and *qPGC11* for low chalkiness in their substitution segments respectively, were used to develop QTL-pyramiding lines. The recipient HJX74 is an *indica* variety widely cultivated in southern China. Substitution segments of the six SSSLs were respectively derived from different donors (Khazar, American Jasmine, Zhong4188, Basmati 370 and HP67) (Additional file 1: Fig. S1 and Additional file 2: Table S1).

All the materials were cultivated in Guangzhou (23°07′N, 113°15′E). The materials were cultivated in two cropping seasons with the FCS from late February to mid-July and the SCS from late July to mid-November per year. Field managements, including fertilization, irrigation, and disease and pest control, were performed by normal agricultural practices in the area (Yang et al. 2021a).

### Genotyping by DNA Markers

During the process of developing QTL-pyramiding lines, individual plants carrying the target QTL combinations were selected by using QTL-linked markers, and then detected the lengths of substitution segments by using all the markers in the substitution segments of SSSLs (Additional file 2: Table S1). The genomic DNA samples of each individual plant were extracted by improved method of CTAB (Stewart and Via 1993). The target DNA segments were amplified by PCR method and the PCR products were separated by 6% denatured polyacrylamide gel electrophoresis and bands visualized by the silver staining method (Yang et al. 2021a).

### Phenotype Investigation and Statistical Analysis

Grain chalkiness is quantified as the PGC, which is equal to the product of the percentage of chalky grains and the percentage of chalky area (Yang et al. 2021a). After seed full maturity, the three plots of each SSSLs and pyramiding lines were harvested respectively. The dry samples of grain from each plot were dehulled and polished, and then the milled head rice per plot was measured three times independently for chalky traits by using the rice quality analyzer SC-E software (Hangzhou Wanshen Testing Technology Co., Ltd. China, www.wseen.com).

The comparison between the data in two groups was conducted using Student’s *t*-test. Dunnett’s *t*-test was used to compare multiple groups with the control group. Least significance range (LSR) test was employed for conducting multiple range tests among multiple sets of data. IBM SPSS 20.0, Microsoft Excel 2016, MapChart 2.3 and GraphPad Prism 8 were employed for statistical analysis, genetic map making and figure making.

### Estimation of Additive Effects and Epistatic Effects

The additive effect of a QTL was estimated by comparing the phenotypic differences in PGC between HJX74 and a target SSSL, and the additive effect of combinations of two- or multiple-loci genotypes was estimated by comparing the phenotypic differences in PGC between HJX74 and relevant pyramiding lines. The estimation of epistatic effect among QTLs for PGC in QTL-pyramiding lines was carried out using the formula, $i=\left({P}_{n}-{P}_{0}\right)-\sum _{i=1}^{n}{(a}_{i})$, where *i* represents an epistasis among the pyramided QTLs, *P*_*n*_ represents a phenotypic value of a pyramiding line carrying *n* of QTLs, *P*_*0*_ represents a phenotypic value of HJX74, *a*_*i*_ (1 ≤ *i* ≤ *n*) represents an additive effect of a single QTL at the *i*th QTL. The significance test of epistatic effects was performed by Student’s *t*-test under null hypothesis (H_0_: *i* = 0) (Tan et al. 2022a).

## Results

### Genotypes and Phenotypes in QTL-Pyramiding Lines

Six SSSLs (22 − 05, 15 − 06, 15 − 08, 03–08, 11 − 09 and HP67-11) were used to develop QTL-pyramiding lines (Additional file 1: Fig. S1 and Additional file 2: Table S1). These SSSLs each carried only one QTL for low chalkiness in their substitute fragments, and were named 1-QTL lines (1QLs). 2-QTL lines (2QLs) were developed by the mutually crossing of the six 1QLs. The two 2QLs which carried one same QTL were crossed to generate 3-QTL lines (3QLs). 4-QTL lines (4QLs) were developed by the crossing of 3QLs and 2QLs or other 3QLs (Additional file 1: Fig S2). Finally, we constructed 17 QTL-pyramiding lines carrying different combinations of QTLs for PGC, including eight 2QLs, five 3QLs and four 4QLs (Additional file 1: Figs. S3-S5 and Additional file 2: Table S2).

**Fig. 1:**
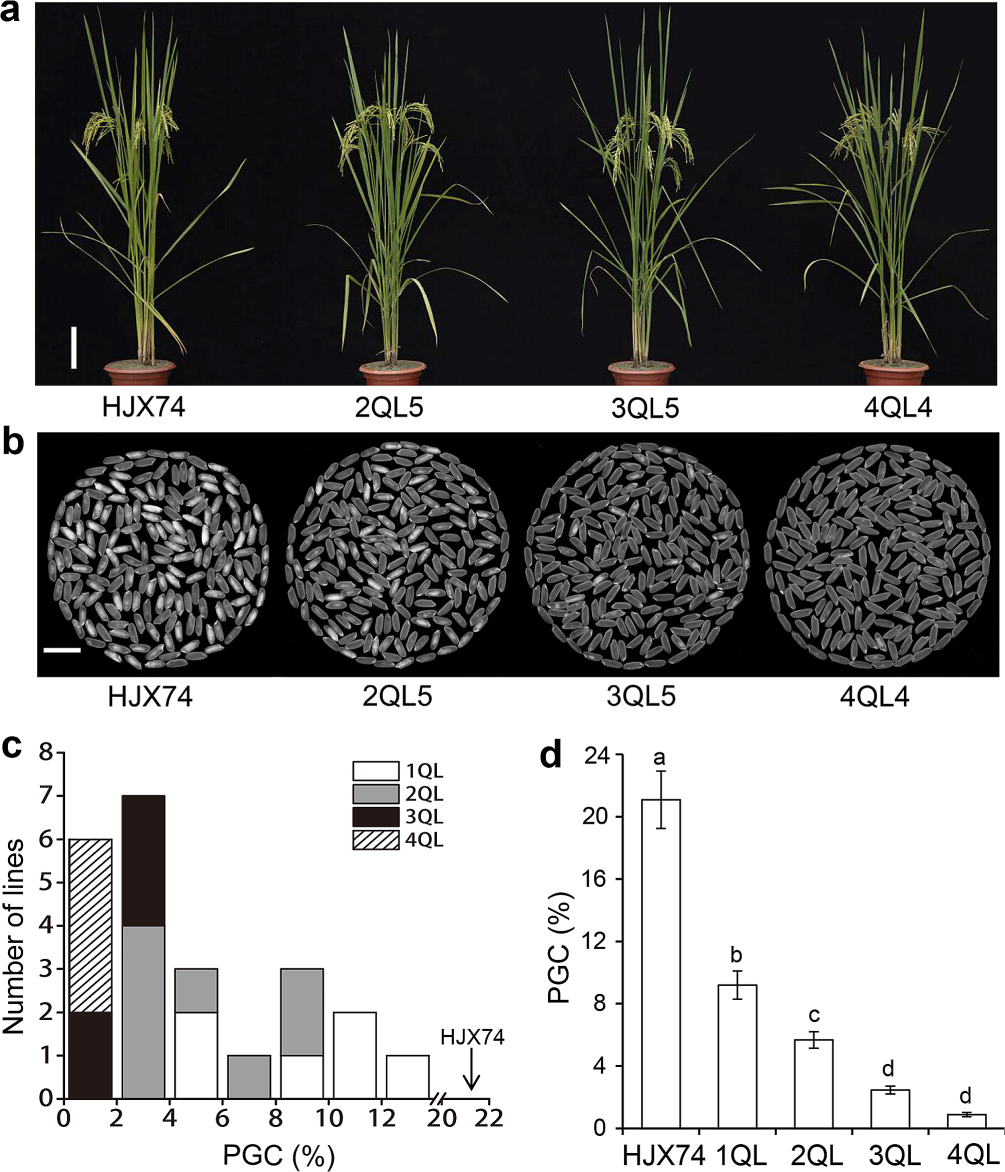
Rice chalkiness of HJX74 and 1QLs to 4QLs. **a**, Plant morphology of HJX74 and the 2QL5, 3QL5 and 4QL4 lines. Bar = 15 cm. **b**, The appearance of head rice of HJX74 and the 2QL5, 3QL5 and 4QL4 lines. Bar = 1 cm. **c**, Frequency distributions of PGC in 1QLs to 4QLs. **d**, PGC in HJX74 and 1QLs to 4QL, Data are given as mean ± S.E. of six cropping seasons. Different lowercase letters denote the significance of difference at the level of *P* ≤ 0.05

The phenotypes of rice chalkiness in the 1QLs to 4QLs were measured in consecutive six cropping seasons. In every cropping season, the chalky traits of the 1QLs to 4QLs were significantly reduced compared to the recipient HJX74 (Additional file 2: Table S3 and Fig. 1). The mean value of PGC in the six 1QLs was 9.22%, with a range of 4.21 to 12.47%, which was significantly lower than 21.11% of HJX74. In the eight 2QLs, the mean value of PGC was 5.43%, with a range of 2.24 to 9.46%, which was 3.78% lower than that in 1QLs. In the five 3QLs, the mean value of PGC was 2.31%, with a range of 1.38 to 3.42%, which was 3.12% lower than that in 2QLs. In the four 4QLs, the mean value of PGC was 0.83%, with a range of 0.52 to 1.08%, which was 1.48% lower than that in 3QLs. (Fig. 1c-d). These results showed that rice chalkiness significantly reduced with the increasing QTLs in the pyramiding lines. All five 3QLs had less than 5.00% PGC in both FCS and SCS, which meet the national grade 2 for good-quality rice. All four 4QLs had less than 2.00% PGC in both FCS and SCS, which meet the national grade 1 for good-quality rice (Additional file 2: Table S3).

### Additive Effects of QTLs on PGC in 1QLs to 4QLs

**Fig. 2:**
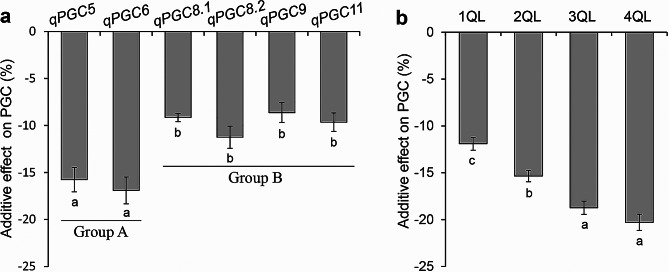
Additive effects of QTLs on PGC. **a**, Additive effects of 6 QTLs on PGC. **b**, Additive effects of QTL combinations in 1QLs to 4QLs. Data are given as mean ± S.E. of six cropping seasons. Different lowercase letters denote the significance of difference at the level of *P* ≤ 0.05

The additive effects of the six QTLs and seventeen combinations with 2–4 QTLs on PGC were calculated in SSSLs and QTL-pyramiding lines (Additional file 2: Table S4). The six QTLs showed great variation in additive effects on PGC, with a range of -8.65 to -17.03%. The additive effects of *qPGC5* and *qPGC6* were − 15.43 and − 17.03% respectively, which were significantly greater than those of the four QTLs, *qPGC8.1*, *qPGC8.2*, *qPGC9* and *qPGC11*, with a range of -8.65 to -11.26%. Therefore, *qPGC5* and *qPGC6* had high additive effect in Group A, while the other four QTLs had low additive effect in Group B (Fig. 2a). The mean value of additive effect in eight combinations with 2 QTLs on PGC was − 15.69%, with a range of -11.66 to -18.87%. The mean value of additive effect in five combinations with 3 QTLs on PGC was − 18.79%, with a range of -17.71 to -19.72%. The mean value of additive effect in four combinations with 4 QTLs on PGC was − 20.31%, with a range of -20.03 to -20.76%. (Fig. 2b, Additional file 2: Table S4). These results showed that the total additive effects of QTL combinations increased with the increase of QTLs in 1QLs to 4QLs.

### Epistasis of Additive by Additive Interaction Among QTLs on PGC in 2QLs to 4QLs

**Fig. 3:**
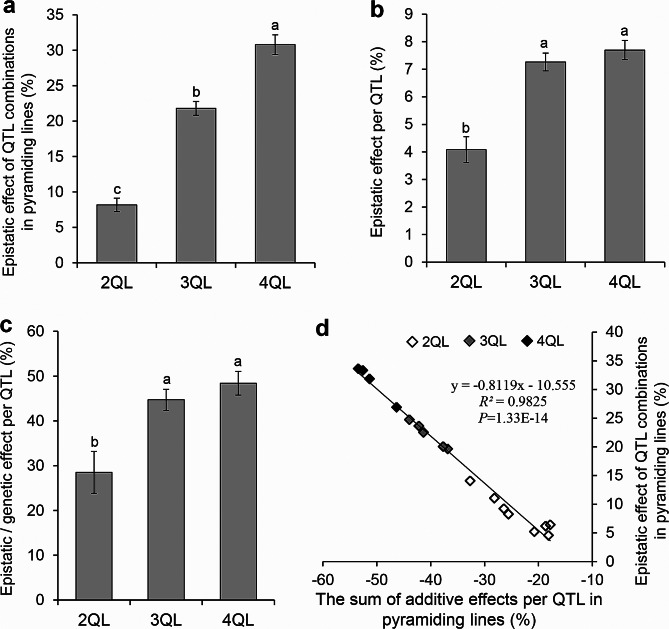
Epistatic effects among QTLs on PGC. **a**, The total epistatic effects of QTL combinations on PGC in 2QLs to 4QLs. **b**, The average epistatic effects per QTL on PGC in 2QLs to 4QLs. **c**, Percentage of epistatic effects in genetic effects for each QTL in 2QLs to 4QLs. **d**, Correlation between sum of additive effects of each QTL and epistatic effects of QTL combinations in pyramiding lines. *R*^*2*^ represents the percentage of x contribution to y variation. Data are given as mean ± S.E. of six cropping seasons. Different lowercase letters denote the significance of difference at the level of *P* ≤ 0.05

Only the epistasis of additive by additive interaction was estimated because of the 1QLs to 4QLs having homozygous genotypes in the same genetic background. In the eight 2QLs, the mean value of epistatic effect of each QTL combination was 8.15%, with a range of 4.57 to 14.08%, with each QTL having an epistatic effect of 4.08%. In the five 3QLs, the mean value of epistatic effect of each QTL combination was 22.13%, with a range of 19.65 to 24.75%, with each QTL having an epistatic effect of 7.38%. In the four 4QLs, the mean value of epistatic effect of each QTL combination was 31.44%, with a range of 26.91 to 33.64%, with each QTL having an epistatic effect of 7.86% (Fig. 3a, b and Additional file 2: Table S5). The above results displayed that the total epistatic effects among QTLs were increased with the increase of QTLs in the pyramiding lines, which were opposite to the direction of additive effects (Fig. 3a, d). The epistatic effects of each QTL accounted for 28.49%, 44.71%, and 48.41% of the genetic effects in 2QLs, 3QLs and 4QLs, respectively (Fig. 3c). Although epistasis existed, the results showed that additivity was still the main component of QTL effects.

To further reveal the relationship between additive effect and epistatic effect, regression correlation analysis between additive effects and epistatic effects of different combinations with 2–4 QTLs was performed in QTL-pyramiding lines. The results showed that the sum of the additive effects of each QTL was significantly positively correlated with the epistatic effects of QTL combinations (*R*² = 0.9825, *P* = 1.33E-14) (Fig. 3d). These results indicated that the higher the sum of additive effect of each QTL, the higher the epistasis of additive-by-additive interaction in QTL combinations.

### *qPGC5* and *qPGC6* Showed Greater Effects to Reduce Chalkiness

**Fig. 4:**
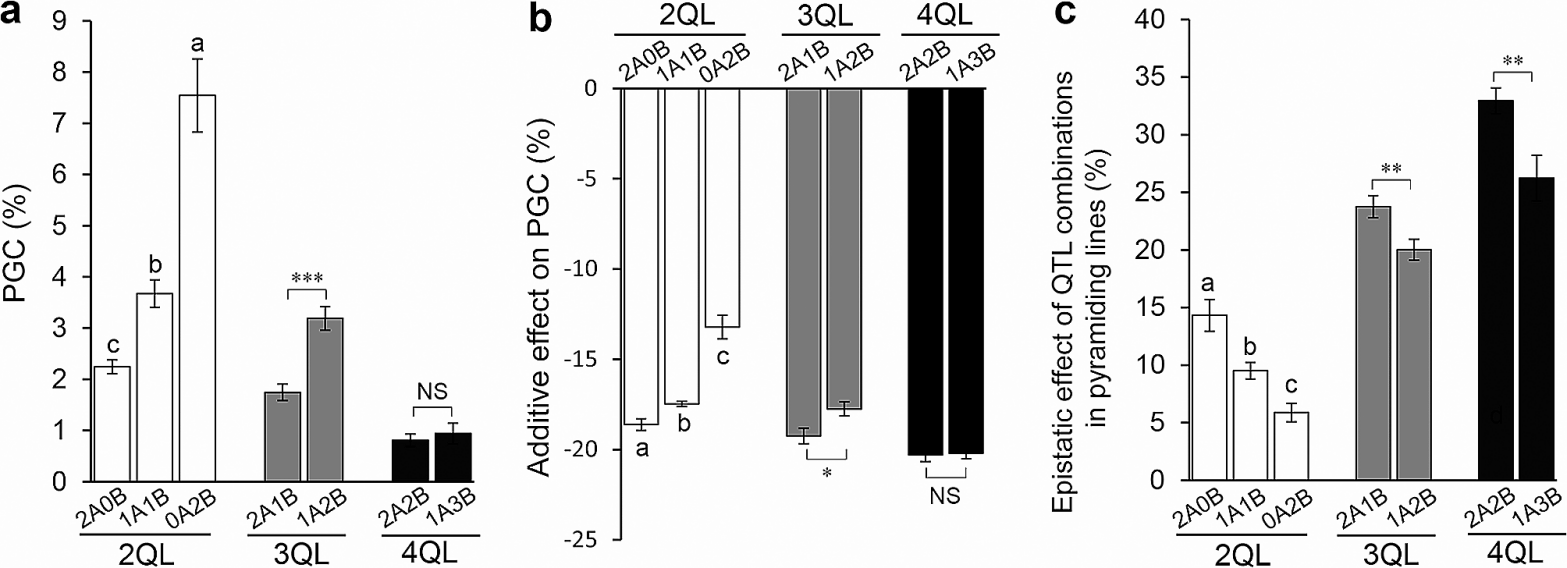
The effects of the QTL combinations with and without *qPGC5* or *qPGC6* on PGC. **a**, PCG in the pyramiding lines with and without *qPGC5* or *qPGC6*. **b**, Additive effects of QTL combinations in the pyramiding lines with and without *qPGC5* or *qPGC6*. **c**, Epistatic effects of QTL combinations in the pyramiding lines with and without *qPGC5* or *qPGC6*. *A* represents *qPGC5* or *qPGC6*, while *B* represents *qPGC8.1*, *qPGC8.2*, *qPGC9* or *qPGC11.* Data are given as mean ± S.E. of six cropping seasons. Different lowercase letters denote the significance of difference at the level of *P* ≤ 0.05. *, ** and *** denote the significant difference at 0.05, 0.01 and 0.001 levels, respectively

According to the number of QTLs of Group A and Group B in QTL combination lines, 17 QTL-combinations can be divided into 7 genotypes. In 2QLs, the average PGC of pyramiding lines with genotypes of 2A0B, 1A1B and 0A2B were 2.24%, 3.68% and 7.55%, respectively, with significant differences between genotypes. In 3QLs, the average PGC of 2A1B-lines was 1.75%, which was significantly lower than the 3.19% of 1A2B-lines. However, in 4QLs, the average PGC of 2A2B-lines and 1A3B-lines respectively was 0.81% and 0.94%, which showed no significant difference (Fig. 4a). For additive effects on PGC, 2A0B, 1A1B and 0A2B of 2QLs were − 18.62%, -17.47% and − 13.22%, respectively, with significant differences between genotypes. 2A1B and 1A2B of 3QLs were − 19.25% and − 17.76%, respectively, with significant difference between genotypes. However, 2A2B and 1A3B of 4QLs were − 20.29% and − 20.21%, respectively, with non-significant difference between genotypes (Fig. 4b). For epistatic effects of QTL combinations, 2A0B, 1A1B and 0A2B of 2QLs were 14.33%, 9.52% and 5.91%, 2A1B and 1A2B of 3QLs were 23.75% and 20.02%, and 2A2B and 1A3B of 4QLs were 32.95% and 26.24%, respectively. There were significant differences between genotypes in all three sets (Fig. 4c). The results displayed that *qPGC5* and *qPGC6* of Group A showed lower PGC, greater additive effect and greater epistatic effect than other QTLs in the pyramiding lines. These findings indicated that *qPGC5* and *qPGC6* showed a greater additive effect in the QTL-pyramiding lines, leading to a more effective reduction in rice chalkiness.

### Influence of Different Cropping Seasons on Rice Chalkiness in QTL-Pyramiding Lines

**Fig. 5:**
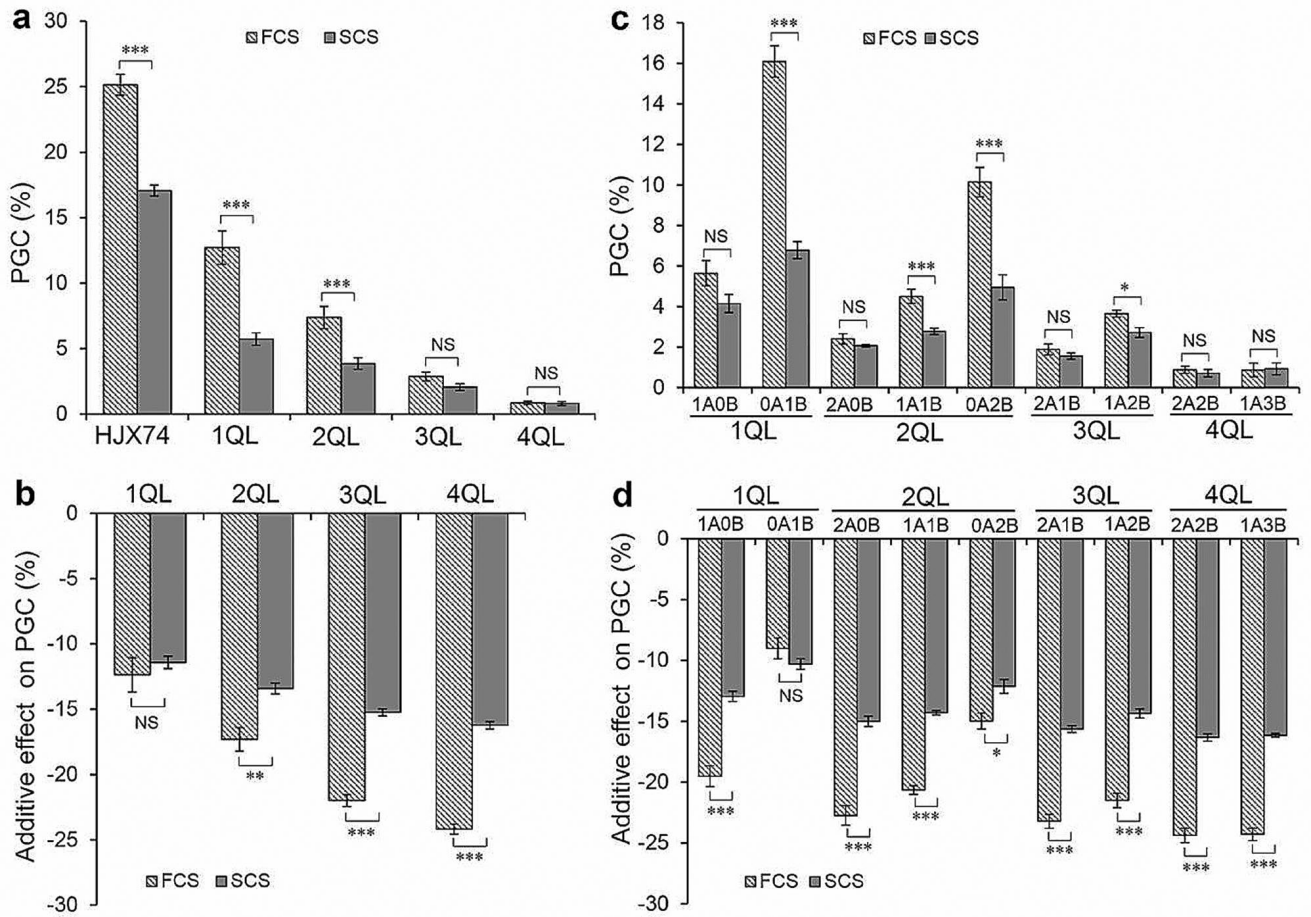
The effects on PGC of HJX74 and 1QLs to 4QLs in FCS and SCS. **a**, PGC of HJX74 and 1QLs to 4QLs in different cropping seasons. **b**, The additive effects of QTL combinations on PGC of 1QLs to 4QLs in different cropping seasons. **c**, PGC of the 9 sets of genotypes with and without *qPGC5* or *qPGC6* in different cropping seasons. **d**, The additive effects of the 9 sets of genotypes with and without *qPGC5* or *qPGC6* in different cropping seasons. *A* represents *qPGC5* or *qPGC6*, while *B* represents *qPGC8.1*, *qPGC8.2*, *qPGC9* or *qPGC11*. *, ** and *** denote the significant difference at 0.05, 0.01 and 0.001 levels, respectively

The temperatures during the rice seed-filling stage in FCS and SCS were significantly different. In the three experimental years, the average values of maximum temperature, minimum temperature and mean temperature were respectively 32.2 °C, 25.9 and 29.0 °C in FCS, and 28.6 °C, 21.0 and 24.9 °C in SCS, which showed the temperature in FCS was higher than that in SCS (Additional file 2: Table S6). To detect the influence of different cropping seasons on rice chalkiness in QTL-pyramiding lines, the rice chalkiness in 1QLs to 4QLs were compared in different cropping seasons. The average PGC of HJX74, 1QLs, 2QLs, 3QLs and 4QLs were 25.14%, 12.61%, 7.37%, 2.61% and 0.86% in FCS, and 17.07%, 5.91%, 3.86%, 2.06% and 0.81% in SCS, respectively. HJX74, 1QLs and 2QLs showed significantly higher PGC in FCS compared to those in SCS, while the PGC of 3QLs and 4QLs showed no significant difference between FCS and SCS (Fig. 5a). These results showed that the differences of PGC between FCS and SCS were weakened with the increase of QTLs in the 1QLs to 4QLs. The average additive effects on PGC of 1QLs, 2QLs, 3QLs and 4QLs were − 12.51%, -17.76%, -22.08% and − 24.28% in the FCS, and − 11.18%, -13.22%, -15.23% and − 16.26% in SCS, respectively. The 2QLs, 3QLs and 4QLs showed greater additive effects in FCS than those in SCS, while the additive effects of 1QLs showed no significant difference between FCS and SCS (Fig. 5b). These findings indicated that the differences of additive effects between FCS and SCS became more significant with the increase of QTLs in the lines with 1- to 4-QTL and the QTL combinations of 3QLs and 4QLs had greater additive effects at HT of FCS, resulting in more effective in reduction of HT-induced chalkiness.

The effects on PGC of 9 sets of genotypes with and without *qPGC5* or *qPGC6* of Group A were analyzed between FCS and SCS. In 1QLs, the PGC in 1A0B-lines showed no significant difference between FCS and SCS, while that in 0A1B-lines showed significant difference. In 2QLs and 3QLs, the PGC in 2A0B-lines and 2A1B-lines showed no significant difference between FCS and SCS, while that in 1A1B-lines, 0A2B-lines and 1A2B-lines showed significantly higher in FCS than that in SCS. In 4QLs, all QTL-pyramiding lines, including 2A2B-lines and 1A3B-lines, showed lower than 1.00% of PGC with no significant difference between FCS and SCS (Fig. 5c). These results indicated that 4QLs had stable lower PGC even in the HT environment of FCS. For additive effects, 1A0B-lines showed significantly higher in FCS than that in SCS, while 0A1B-lines showed no significant difference. This was the results why the PGC in 1A0B-lines showed no significant difference between FCS and SCS, while that in 0A1B-lines showed significant difference. Among 7 genotypes of 2QLs to 4QLs, the additive effects showed significantly higher in FCS compared to those in SCS, with 2A-genotypes having the highest additive effects (Fig. 5d). These results indicated that the HT of FCS enhanced the additive effects of *qPGC5* and *qPGC6*, leading to higher total additive effects in pyramiding lines with the two QTLs of Group A to reduce HT-induced chalkiness.

## Discussion

### Pyramiding of QTLs for Low Chalkiness Can Reduce Rice Chalkiness to the Best Quality Level

Chalkiness is a complex quantitative trait and is susceptible to environmental factors. It was found that the proportion of *indica* varieties meeting the grade 3 national standard of rice quality for rice chalkiness was lower than that of other rice quality traits in China (Feng et al. 2017). Therefore, reducing chalkiness in rice has become the main goal of high-quality rice breeding. Although lots of QTLs for rice chalkiness have been mapped in rice genome, few of them have been used in improvement of rice quality by QTL pyramiding (Fan et al. 2022; Zhao et al. 2022). In previous studies, we identified six QTLs, *qPGC5*, *qPGC6*, *qPGC8.1*, *qPGC8.2*, *qPGC9* and *qPGC11*, for chalkiness by using the SSSLs of HJX74 genetic background (Yang et al. 2021a, b, 2022). In the present research, we used the six QTLs to develop pyramiding lines with different QTL combinations. The results showed that the chalkiness of rice in 1QLs to 4QLs decreased with the increase of pyramided QTLs. The average PGC in 3QLs was 2.31%, meeting the national grade 2 for good-quality rice. The average PGC in 4QLs was 0.83%, meeting the national grade 1 for good-quality rice (Fig. 1c-d and Additional file 2: Table S3). These results showed that the pyramiding lines with four of the QTLs significantly decreased the chalkiness of rice and reached the best quality level. Our research demonstrates that the good-quality rice varieties with low chalkiness at the best quality level can be developed by pyramiding QTLs for low chalkiness.

In general, there is a trade-off effect between grain quality and grain yield in rice cultivars accessions. High-quality rice cultivars are usually negatively correlated with grain yield (Wang et al. 2012a; Ren et al. 2023). However, in our previous studies, the six SSSLs, carrying *qPGC5*, *qPGC6*, *qPGC8.1*, *qPGC8.2*, *qPGC9* and *qPGC11* for low grain chalkiness respectively, exhibited similar yield traits to the recipient HJX74 (Yang et al. 2021a, b, 2022). The six QTLs significantly reduced grain chalkiness without sacrificing its yield. Therefore, the trade-off effect between grain quality and yield of the QTL-pyramiding lines needs further observation.

### Pyramiding of QTLs for Low Chalkiness Can Reduce Rice Chalkiness Even in the HT Environment of FCS

With the gradual increase of the global warming, HT accelerates formation of rice chalkiness (Nevame et al. 2018; Zhao et al. 2022). In the double-cropping rice area of southern China, the emergence of HT during the rice seed-filling stage in FCS results in higher chalkiness occurrence compare to that in SCS (Additional file 2: Table S6) (Cheng et al. 2005, 2019; Yang et al. 2021a). In previous studies, we have learned that the six QTLs for chalkiness have different response to HT. *qPGC9* and *qPGC11* were sensitive to HT of FCS, which weaken their additive effects on chalkiness under HT (Yang et al. 2021a). The additive effects of *qPGC8.1* and *qPGC8.2* were not influenced by HT (Yang et al. 2021b). *qPGC5* and *qPGC6* showed higher additive effects enhanced by HT (Yang et al. 2022). In the present study, based on the magnitude of the additive effects on chalkiness, the six QTLs were divided into two groups, in which *qPGC5* and *qPGC6* showed higher additive effects in Group A, and other four QTLs showed lower additive effects in Group B (Fig. 2). It was found that there were significant differences in the PGC between FCS and SCS in recipient HJX74, 1QLs, and 2QLs, while there were no significant differences in 3QLs and 4QLs (Fig. 5). Comparing PGC in the 2QLs to 4QLs with and without *qPGC5* or *qPGC6*, it was displayed that *qPGC5* and *qPGC6* showed lower PGC, greater additive effects and greater epistatic effects than other QTLs in the pyramiding lines (Fig. 4). In addition, the HT of FCS enhanced the additive effects of *qPGC5* and *qPGC6*, thereby reducing the HT-induced chalkiness (Fig. 5). Therefore, *qPGC5* and *qPGC6* have stronger ability to reduce rice chalkiness, particularly in the HT environment of FCS.

### Pyramiding of Multiple QTLs Is an Effective Way for Improving Complex Traits

It is known that most traits are complex traits in rice, which are controlled by multiple QTLs (Ashikari and Matsuoka 2006; Zhang 2021). Some successful cases for improving important agronomic traits in rice by introgressing favorable alleles via marker-assisted selection have been reported (Liu et al. 2006; Wang et al. 2012b; Hur et al. 2016; Zeng et al. 2017; Xu et al. 2021; Zhang et al. 2021). However, there are few of cases of pyramiding multiple QTLs to improve complex traits were reported in rice breeding (Kumar et al. 2018; Zhang 2021; Tan et al. 2022a; Ye et al. 2022). Recently, the complex trait of high stigma exsertion rate (SER) was rebuilt by using eleven QTLs for SER. The twenty-nine 2QLs to 6QLs were developed from the SSSLs carrying QTLs for SER. The SER of the pyramiding lines increased with increase of pyramided QTLs and the 5QLs and 6QLs showed the high SER comparable to that of wild rice (Tan et al. 2020, 2021, 2022a, b). The rebuilding of the high-SER trait provides an example for improving complex traits in rice breeding. Rice chalkiness is another complex trait. Improvement of rice chalkiness is challenge for rice breeders, due to the complex heritability of chalkiness and its susceptibility to environmental factors (Zhao et al. 2016; Feng et al. 2017; Nevame et al. 2018). In this study, we successfully developed the QTL-pyramiding lines with low chalkiness at the best quality level even in the HT environment of FCS by pyramiding 4 QTLs for low chalkiness. Our results provide an example for reducing chalkiness in rice breeding.

Although there may be a large number of QTLs for each complex trait in a wide range of genetic resources, the number of QTLs controlling a single complex trait in each line is limited (Tan et al. 2020, 2021, 2022a, b). Due to the epistasis existing among QTLs, the QTL effects are usually less-than-additive (Eshed and Zamir 1996; Tan et al. 2022a). Therefore, although a large number of QTLs for each complex trait in a wide range of genetic resources have been identified, several QTLs are enough to rebuild a complex trait with expected target. In our previous studies, eighteen QTLs for SER were identified from different rice accessions, however, the combinations of 5–6 QTLs of them are enough to rebuild high-SER trait comparable to that of wild rice (Tan et al. 2020, 2021, 2022a, b). In the present study, the epistatic effect among QTLs on PGC was in opposite direction of additive effect, which caused the effect of the combination of QTLs was less than the sum of the additive effects of the corresponding individual QTLs (Figs. 2 and 3). The correlation analysis indicated that the higher the sum of additive effects of each QTL, the higher the epistasis of additive-by-additive interaction in QTL combinations (Fig. 3d). Therefore, although we have identified 6 QTLs for low chalkiness from different donors (Yang et al. 2021a, b, 2022), the combinations of 4 QTLs are enough to develop the lines with low chalkiness at the best quality level even in the HT environment of FCS. Complex traits can be improved by pyramiding several QTLs.

## Conclusion

Rice chalkiness in the 1QLs to 4QLs with the genetic background of HJX74 decreased with the increase of pyramided QTLs. The 4QLs significantly reduced the chalkiness of rice and reached the best quality level. *qPGC5* and *qPGC6* have a stronger ability to reduce rice chalkiness, especially in the HT environment of FCS. New rice varieties with low chalkiness can be developed by pyramiding low chalkiness QTLs. Pyramiding of low chalkiness QTLs is an effective way to reduce chalkiness in rice.

### Electronic Supplementary Material

Below is the link to the electronic supplementary material.

**Supplementary Material 1**: **Fig. S1** QTLs for PGC and their positions in the substitution segments in SSSLs. **Fig. S2** Development of pyramiding lines with different QTL combinations for PGC. **Fig. S3** QTLs for PGC and their substitution segments in 2QLs. **Fig. S4** QTLs for PGC and their substitution segments in 3QLs. **Fig. S5** QTLs for PGC and their substitution segments in 4QLs

**Supplementary Material 2**: **Table S1** QTLs for PGC and their position in the substitution segments in SSSLs. **Table S2** Substitution segments carrying QTLs for PGC in pyramiding lines. **Table S3** The phenotypes of rice chalkiness in 1QLs to 4QLs. **Table S4** Additive effects of QTL combinations on PGC in 1QLs to 4QLs. **Table S5** Epistatic effects of QTL combinations on PGC in pyramiding lines. **Table S6** Average temperatures of 30 days after rice flowering in different cropping seasons

## Data Availability

All of the datasets supporting this study are included in the published article and its supplementary information files.

## Abbreviations

1QLs: 1-QTL lines
2QLs: 2-QTL lines
3QLs: 3-QTL lines
4QLs: 4-QTL lines
FCS: First cropping season
HT: High temperature
HJX74: Huajingxian 74
PGC: Percentage of grain chalkiness
SCS: Second cropping season
SER: Stigma exsertion rate
SSSL: Single-segment substitution line
