# Supplementary material for: Pyramiding of Low Chalkiness QTLs Is an Effective Way to Reduce Rice Chalkiness
Source: Rice (N Y). 2024 Jan 8;17:4. doi: 10.1186/s12284-023-00680-x (PMC10772014; doi:10.1186/s12284-023-00680-x)
Supplement: Supplementary file 1 — Supplementary Material 1: Fig. S1 QTLs for PGC and their positions in the substitution segments in SSSLs. Fig. S2 Development of pyramiding lines with different QTL combinations for PGC. Fig. S3 QTLs for PGC and their substitution segments in 2QLs. Fig. S4 QTLs for PGC and their substitution segments in 3QLs. Fig. S5 QTLs for PGC and their substitution segments in 4QLs [file 12284_2023_680_MOESM1_ESM.pdf]

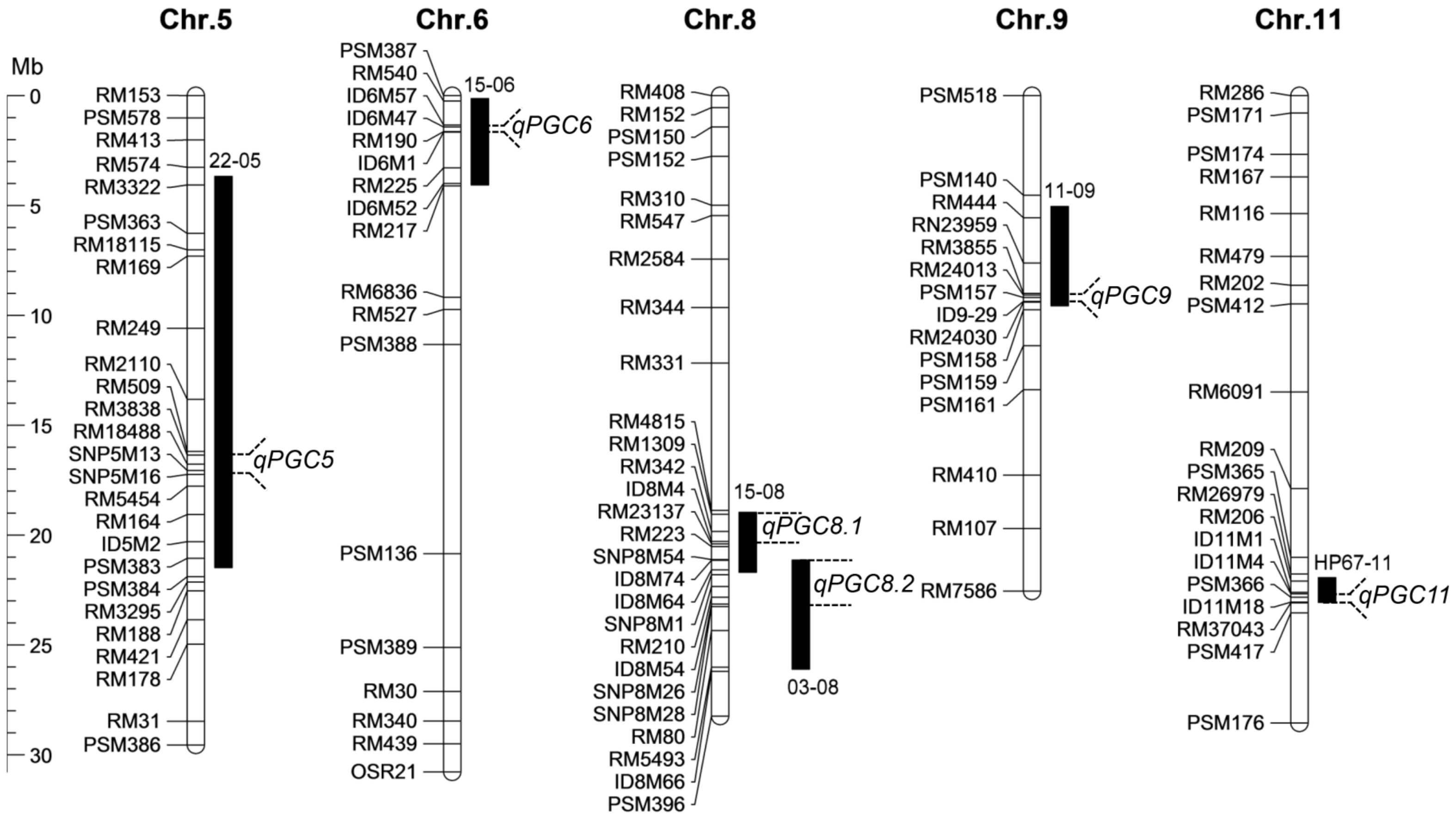

Fig. S1 QTLs for PGC and their positions in the substitution segments in SSSLs.

PGC, percentage of grain chalkiness. SSSL, single-segment substitution line.

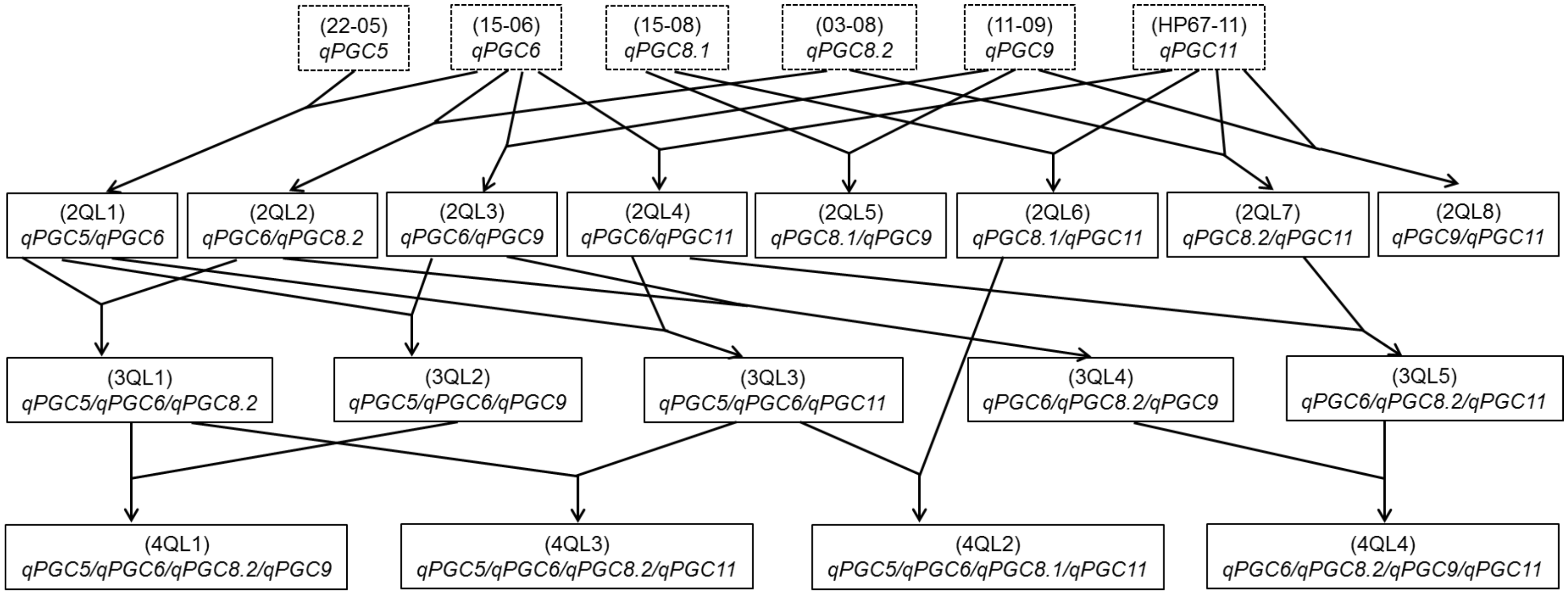

Fig. S2 Development of pyramiding lines with different QTL combinations for PGC. The names in dotted boxes are the parents of pyramiding lines. The names in solid boxes are the developed pyramiding lines.

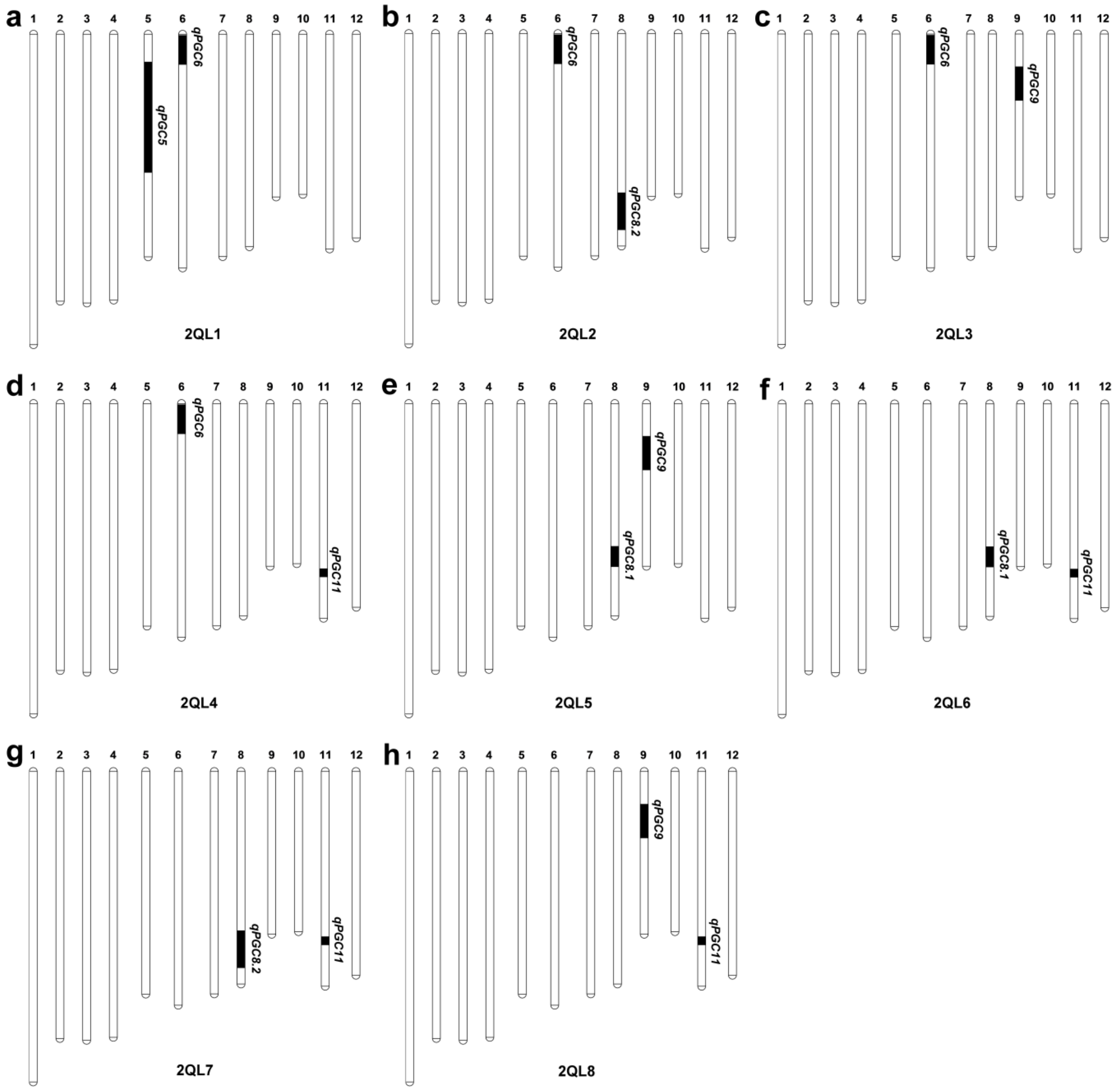

Fig. S3 QTLs for PGC and their substitution segments in 2QLs.

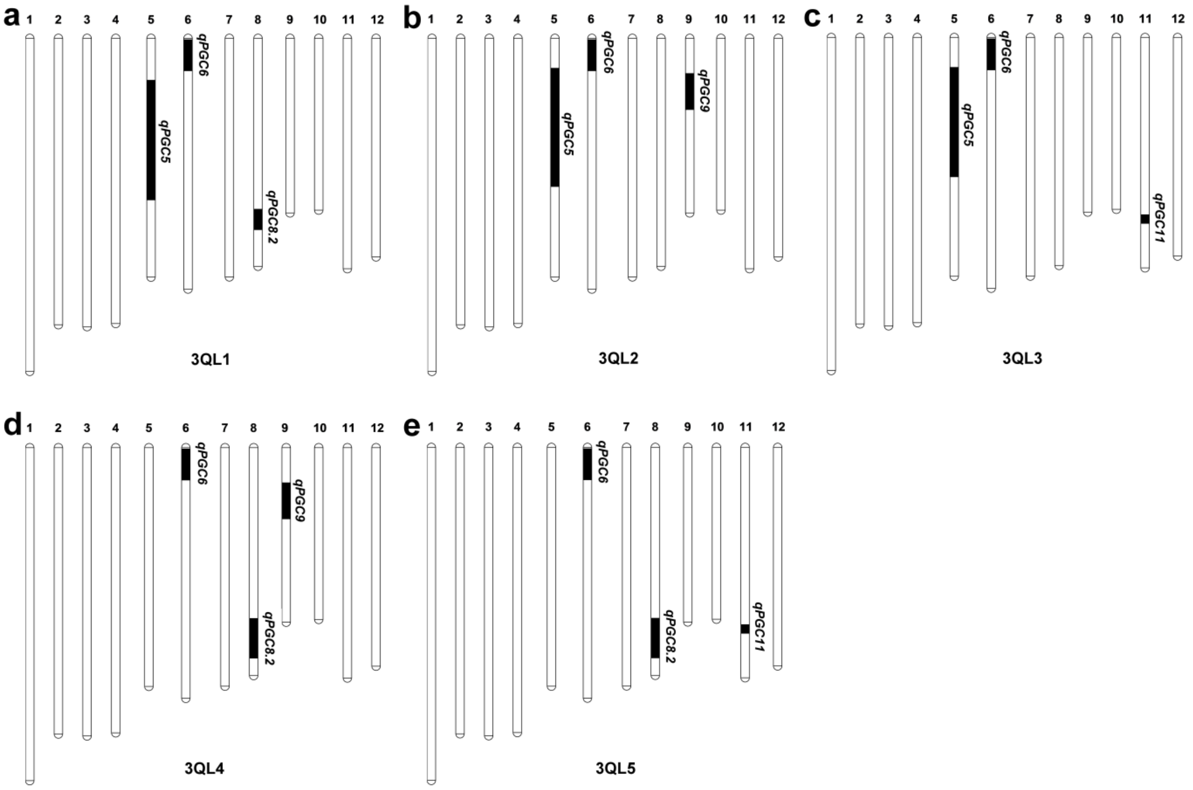

Fig. S4 QTLs for PGC and their substitution segments in 3QLs.

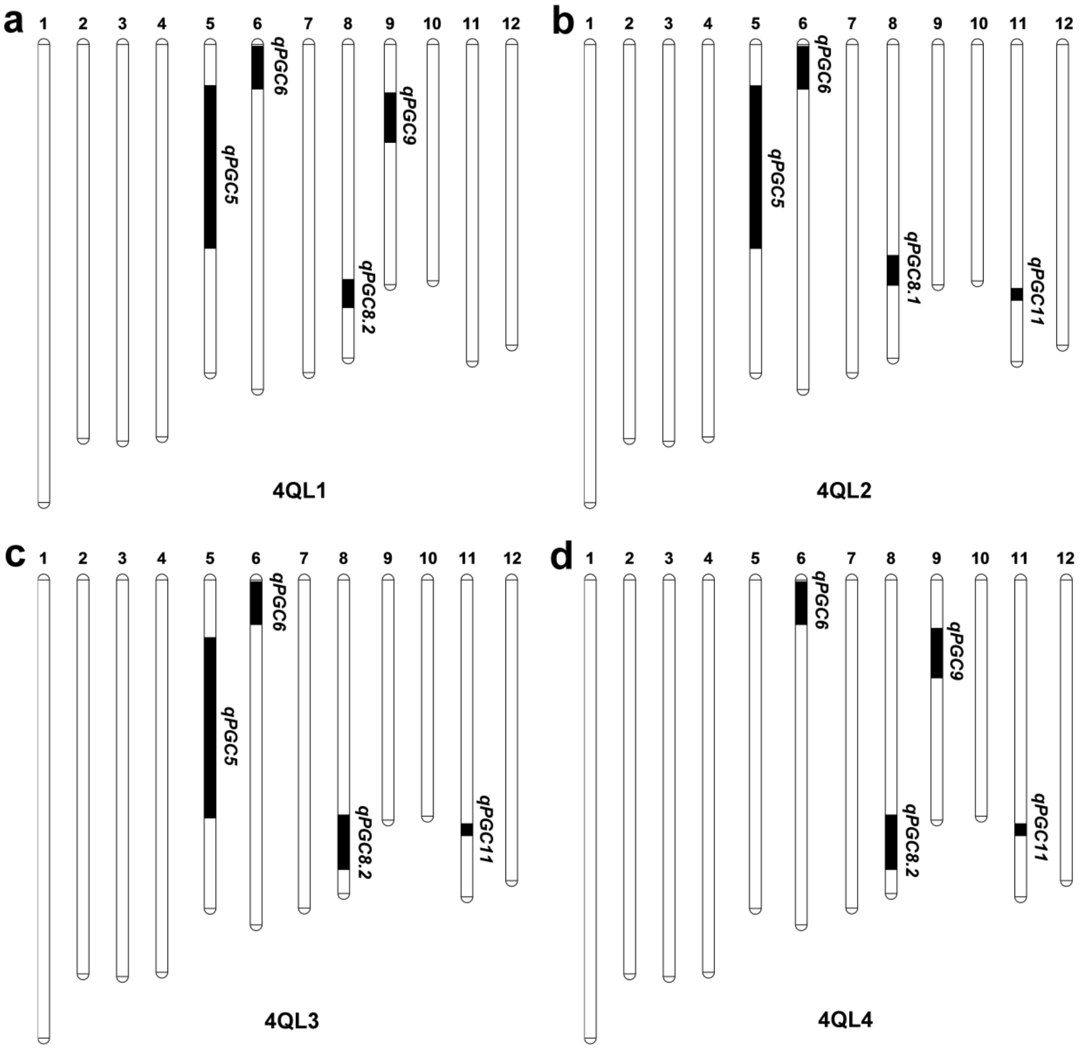

Fig. S5 QTLs for PGC and their substitution segments in 4QLs.
